# A randomized clinical trial compared the effect of intra-alveolar 0.2 % Chlorohexidine bio-adhesive gel versus 0.12% Chlorohexidine rinse in reducing alveolar osteitis following molar teeth extractions

**DOI:** 10.4317/medoral.19932

**Published:** 2014-12-05

**Authors:** Nedal-Abdullah Abu-Mostafa, Abdullah Alqahtani, Mohammed Abu-Hasna, Ahmed Alhokail, Ammar Aladsani

**Affiliations:** 1 BDS. MSc.Lecturer in Oral and Maxillofacial Surgery. Riyadh Colleges of Dentistry and Pharmacy, Oral and Maxillofacial Surgery and Diagnostic science Department, Dental Hospital (Munessya) Riyadh, Kingdom of Saudi Arabia; 2 BDS. General Dental Practitioner. Riyadh Colleges of Dentistry and Pharmacy, Oral and Maxillofacial Surgery and Diagnostic Science Department, Dental Hospital (Munessya) Riyadh, Kingdom of Saudi Arabia

## Abstract

Objectives: To evaluate socket healing, incidence of acute alveolar ostieitis (AO) and associated pain following single molar tooth extraction in patients who receive intra-alveolar 0.2% chlorhexidine (CHX) gel, and those who rinsed with 0.12 % CHX rinse. Study Design: A prospective randomized clinical trial was conducted on two parallel groups of patients. Group1 (141 patients): Rinsed with 0.12 % CHX rinse from the second postoperative day, two times daily for a week. Group2 (160 patients): Who had direct intra-alveolar application of 0.2% CHX gel and day 3 post-operatively. The socket was evaluated 3 and 7 day post operatively for the presence of AO by checking probing tenderness in the socket, empty socket, food debris, halitosis and pain assessment by VAS. Results: Forty-eight AO cases were diagnosed out of 301 extractions (15.9%). In Group1, 25 cases were found (17.7%) while 23 cases were found in Group2 (14.4%). The difference was not statistically significant (p=0.428). Presence of empty socket and food debris in Group1 were higher than in Group2 but the difference was not statistically significant (*p*= 0.390 & *p* = 0.415). Occurrence of halitosis in Group2 was more than Group1, but the difference was not significant (*p*= 0.440). Statistical significance was found between AO in extraction done by root separation (29%) and those routinely extracted (12.3 %) (*p*=0.001). Conclusions: Postoperative evaluation of molar extraction sockets that received direct intra-alveolar application of 0.2% CHX gel showed insignificant less occurrence of AO when compared with 0.12 % CHX rinse.

** Key words:**Chlorhexidine rinse, bio adhesive gel, alveolarosteitis, dry socket, molar teeth extraction, post-extraction socket.

## Introduction

Alveolar Osteitis (AO) commonly known as “dry socket” remains a common post-extraction complication, resulting in severe pain and discomfort. The most recent definition of AO is “postoperative pain in and around the extraction site, which increases in severity at any time between 1 and 3 days after the extraction accompanied by a partially or totally disintegrated blood clot within the alveolar socket with or without halitosis.” (1) 

AO is diagnosed between 2nd and 4th postoperative days when patients complain of a painful extraction socket and which on clinical examination usually reveals empty socket or disintegrated clot with exposed bone and fetid odor (2-5).

The average rate of AO for dental extractions is variable. Jaafar and Nor (6) found AO in 3% to 4% of dental extractions and a literature reports a range from 1 – 30 % (7). The highest incidence, (20-30%) generally occurs following the surgical extraction of impacted third molars (8).

Risk factors for AO mentioned in the literature include; traumatic surgery, remaining tooth fragment (9,10), smoking, oral contraceptives, advanced age, female gender, immunosuppression (7) and lack of dentist experience which is associated with higher trauma during extraction (11). Bacterial infection is a major risk as the frequency of AO increases in patients with poor oral hygiene, preexisting local infection such as periocoronitis and advanced periodontal disease (12).

Fibrinolysis and bacteria are the main etiological theories on AO (13). Birn H (9) suggested that the etiology of AO is an increased local fibrinolysis leading to disintegration of the clot. The fibrinolysis results from activation of plasminogen pathway, which can be initiated by direct (physiologic) or indirect (non-physiologic) activator substances. Direct activators are released following trauma to the alveolar bone cells while indirect activators are stimulated by bacteria.

Since the role of bacteria in this process is proven, the most effective method for reducing AO has been through the use of agents that systematically or topically reduce the oral bacteria within the wound (2,8).

Systemic and topical antibiotics such as topical tetracycline have been proposed and used for the prevention of AO (14). Antibiotics could be expensive, may create resistance, and their efficiency in the prevention of AO has been questioned by Ritzau *et al*. (15) who did not find any preventive effect of a single dose of metronidazole in the development of AO. Delilbasi *et al*. (16) recommended using chlorhexidine solution with a lactamase inhibitor– containing antibiotic to enhance its effectiveness for the prevention of alveolar osteitis.

Some measures were suggested in the literature for the prevention of AO including washing with saline solution, eugenol dressings to provide relief, anti-fibrinolytic agents and tranexamic acid (3,7).

Chlorhexidine (CHX) is a biguanide antiseptic agent often used as an active ingredient in mouthwash designed to reduce dental plaque and oral bacteria population. It has been shown to have an immediate bactericidal action and a prolonged bacteriostatic action due to adsorption onto the pellicle-coatedenamel surface (17). Since, rinsing with CHX is known to reduce oral microbe population; several studies have reported that the pre- and postoperative use of 0.12% CHX decreases the frequency of AO after mandibular third molar removal (8,18,19). Sridhar *et al* (20) recommended that patients could use 0.2% CHX perioperatively (twice daily, 1 day before and 7 days after the surgical extractions) for the prevention of alveolar osteitis.

Adverse reactions to CHX mouthwash have been documented in the literature and these include altered taste sensation, the bad taste of the solution and staining of dentures, tongue, gingiva, and restorations in addition to numbness and stomach upsets (16,21). These adverse reactions are not observed in patients who used CHX bio-adhesive gel. Bio-adhesive properties of the gel reportedly produce more direct action and prolong the time of the CHX treatment that is more efficient against AO (4,22,23).

Daly *et al*. in 2012 (24) concluded after a meta-analysis study of 21 trails, that perioperative rinsing with 0.12% and 0.2% chlorhexidine gluconate or applying CHX gel in the socket post-extraction are moderately evidenced to be beneficial in preventing AO. They recommended comparative studies of rinsing with CHX and application of intra-socket CHX gel to prevent dry socket. The recommended trials are in general dental practice settings with teeth other than third molars and including non-surgical extractions.

The objective of this study was to evaluate socket healing, the incidence of acute alveolar ostieitis (AO) and associated pain following extraction of molar teeth in patients who receive direct intra-alveolar application of 0.2% CHX gel, and those who receive 0.12 % CHX mouthwash.

## Material and Methods

A prospective randomized experimental parallel clinical trial was carried out on three hundred-one, male and female patients who underwent tooth extraction. For every patient, single extraction was done of upper or lower molar tooth, either routinely or by or root separation. The study involved patients treated from November 2012 to April 2013. Extractions were performed by dental interns or dental students under supervision of surgery instructors in the Colleges Clinics. Inclusion in the study were patients with upper or lower molar teeth indicated for extraction. Exclusion criteria included patients with uncontrolled systemic diseases, epinephrine contraindications, allergy to CHX, Lidocaine and Ibuprofen, pregnant women, breast feeding women and those who were using oral contraceptives. Other exclusion criteria were presence of acute infection, cystic lesions; traumatic extraction with fractured alveolar bone, extraction requiring bone reduction and extractions lasted that lasted more than 30 minutes.

This study followed the Declaration of Helsinki on medical protocol and ethics, and the approval of Ethics Committee of the institute was obtained. The registration number of this study in the Research Center of the institution was (IRP/2012/180).

All patients were informed about the objectives of the study and informed consent had to be signed. All required information was documented in the questionnaire paper regarding name, age, gender, mobile number, file number, smoking, medical condition, tooth indicated for extraction, preoperative pain and halitosis.

Teeth extractions were done under local anesthesia, 2% Lidocaine with 1:80,000 epinephrine. Anesthesia for upper molars was achieved by infiltration, while lower molars were anesthetized by combination of inferior alveolar nerve block and buccal infiltration. Simple extractions were done by elevators and forceps, while root separations were done using a surgical hand piece and burs with normal saline irrigation.

Patients were divided randomly into two parallel groups by asking them to choice one card out of two in which the group number was written on its back. Group1: All patients received a bottle of 0.12% CHX mouthwash (Peridex, Oral Rinse, 3M, ESPE, USA) to start using it on the second day of extraction twice daily for 7 days. Group2: After performing tooth extraction, CHX 0.2% bioadhesive gel (Elugel, 40 ml gel tube, Pierre Fabre Oral Care, Boulogne, Paris, France) was applied into the extraction socket. Postoperative instructions were given for all patients in addition to the prescription of Ibuprofen (Brufen, Hamol Limited, Nottingham, England) 600mg every 8 hours for 3 days.

Patients were followed in the third postoperative day. Reevaluation included tenderness with probing the socket, empty socket, food debris, halitosis and assessment of pain by (VAS) from (0, 1, 2,…10). Score 0 representing no pain, 10 representing severe pain. At the end of the visit, CHX 0.2% bioadhesive gel was applied again into the extraction socket for patients of Group 2. Reevaluation was repeated in the seventh postoperative day including the same evaluation points of the third day.

Acute alveolar ostieitis, (dry socket) was diagnosed if the patient presented between the 2nd and 4th days with pain or tenderness in the socket with probing, empty socket and food debris with or without halitosis.

Frequencies and percentages were calculated for qualitative data (SPSS software version.19). Chi-square test was applied to compare both subgroups.

## Results

Three hundred-eleven patients participated in the study; ten of whom were eventually excluded because they did not use CHX rinse twice daily for a week. The first group comprised patients who received CHX rinse were 141 patients (46.8%). The second group of 160 patients had application CHX gel (53.2%). There were 48 (15.9%) AO cases out of the total number of cases. Twenty-five cases (17.7%) were in the rinse group and 23 cases (14.4%) in in the gel group. The difference was not statistically significant (*p*=0.428).

Two hundred and thirty six patients were males and sixty five were females. AO is more common in Females (23.1%) than males (14%). Eighty eight patients were smokers, AO developed in 17 cases (19.3%). None smoker patients were 213 and AO developed in 31 cases (14.6%). Smoker patients had slightly higher percentage of AO but it was statistically not significant (*p*=0.304).

Thirty four cases of AO occurred in the mandible (70.8%) while 14 cases were in the maxilla (29.2%). The difference was not statistically significant (*p*=0.445). ( Table 1).

**Table 1:**
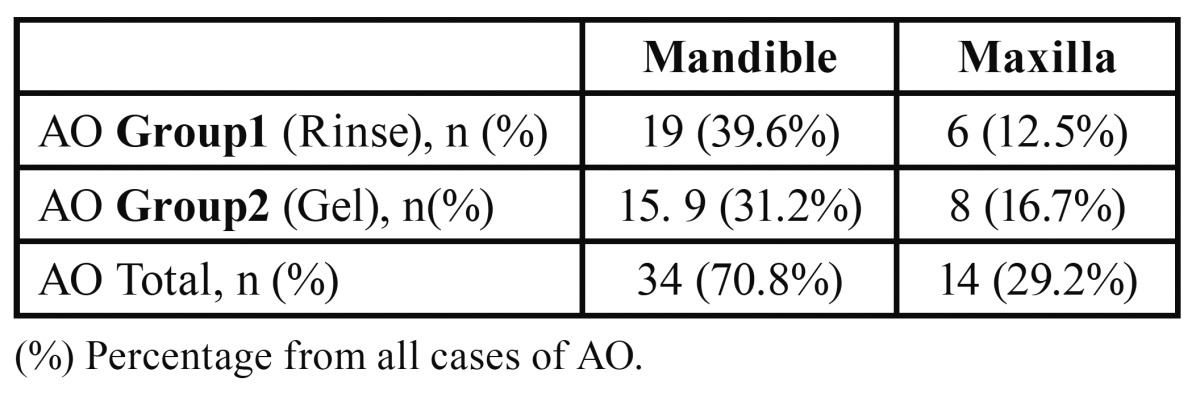
Distribution of AO encountered in Maxilla and Mandible.

The percentage of empty socket in the rinse group was higher than in the gel group but the difference was not statistically significant (*p*=0.390). Incidence of food debris accumulation found in the sockets of the rinse group was higher than the gel group but it was also not statistically significant (*p*=0.415). In contrast, the gel group showed higher incidence of halitosis when compared to the rinse group. Also the difference was not significant (*p*=0.440) ( Table 2).

**Table 2:**
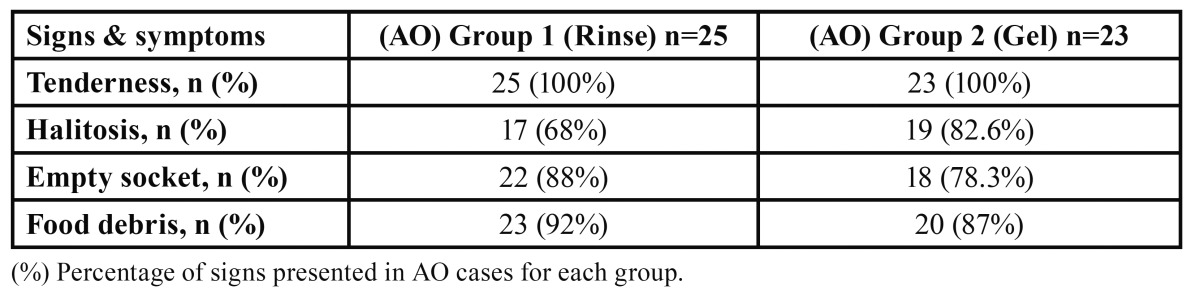
Incidence of the signs and symptoms presented in AO cases in each group.

There was a statistically significant relation between AO and extraction technique used (*p*=0.001). AO was more likely to associate root separation and less likely in simple extraction ( Table 3).

**Table 3:**
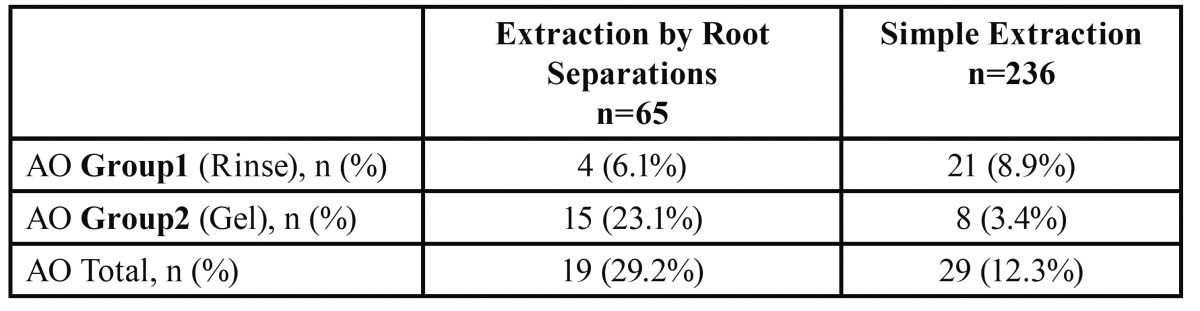
Percentages of AO in relation to the type of extraction performed.

There was no significant difference between the two groups of patients regarding incidence of pain score according to (VAS) ( Table 4).

**Table 4:**
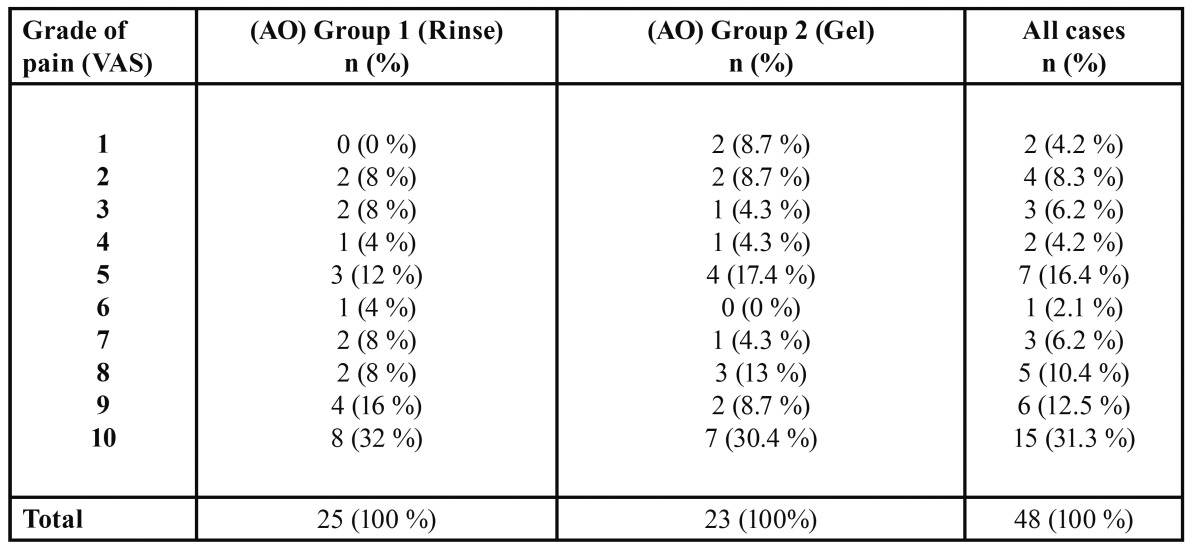
Incidence of pain grades presented in each group.

## Discussion

Literature review revealed several trails which assessed the efficiency of CHX gel in the reduction of AO. Fotos *et al*. (22) carried out a study on patients with mandibular third molar extraction. The patients who received CHX intra-alveolar dressings exhibited a significant reduction in postoperative discomfort when compared with saline solution rinse of control sites (*p* less than 0.005). They pointed out that CHX holds promise as an intra-alveolar antimicrobial medicament for the reduction of postoperative AO.

In 2006 Torres *et al*. (3) concluded that the bio-adhesive 0.2% CHX gel, applied only once after the extraction of impacted third molars, reduced the incidence of alveoli tis. Later, Torres *et al*. (23) did a randomized, double-blind study on 103 patients divided into two groups. In the first group, 0.2% CHX bio-adhesive gel was applied only once post extraction to the socket while in the other group, placebo gel was applied. In the CHX group, the incidence of AO was 11% while the other group was 30%. The reduction in the incidence of AO was significant (*P* = 0.019). Intra-alveolar CHX gel may thus prove to be a good prophylactic agent for AO.

Babar *et al*. (5) in 2012 found that alveolar osteitis developed in 28% following lower third molar surgical extraction, but the percentage of alveolar osteitis was reduced to 8% when intra-alveolar 0.2% CHX bio-adhesive gel was placed for one time after extraction. The difference was significant statistically (*p*=0.017). They suggested that intra-alveolar CHX gel should be considered as a good prophylactic agent for this condition.

A recent study was done by Haraji *et al*. in 2013 (25). They found that a gelatin sponge dressing saturated in 0.2% CHX gel was effective in reducing the incidence of AO following surgical extraction of impacted third molars when compared to dry dressing which was packed as the placebo in the contra lateral sockets. The AO average of CHX side and placebo side was 11.3%, 32.6% respectively. The difference was statically significant (*P* ≤ .001).

Hita-Iglesias *et al*. (4) made a comparison between the effectiveness of the two forms of CHX, gel and rinse, in reducing AO. The study was on 70 patients subjected to surgical extraction of lower wisdom teeth. The first group of patients applied bioadhesive CHX gel on wound twice a day during the first postoperative week. The other group used CHX mouthwash twice a day for a week post operatively. The extraction sockets were evaluated on day 3 and day 7. In the gel group, AO presented in 7.5% of cases, while in the mouthwash group, 25% cases had AO. The difference was statistically significant, (*P* =.040). Additionally, they found insignificant statistical differences in AO incidence in groups of smokers and nonsmokers.

Our findings agree with these of Hita-Iglesias *et al*. (4)as the incidence of AO with patients who used CHX mouth wash in the post-operative days was higher than the patients who received intra-alveolar CHX gel. The difference was not statistically significant (*p*=0.428). Moreover we found insignificant difference in AO incidence between smokers and nonsmokers (*p*=0.428).

Regarding signs and symptoms of AO, we documented the incidence of each of them. The percentages of cases associated with empty socket and food debris in CHX rinse group were 88% and 92% respectively. However the gel group showed slightly decreased percentages of empty socket (78.3%) and food debris (87%). The direct action of CHX bioadhesive gel may have a limited action as filler in the socket within the few hours after extraction causing a decrease in food accumulation and proliferation of bacteria. Halitosis was present in 68% of cases in the rinse group and in 82.6% of the gel group. It could be explained by the localized antibacterial action of CHX gel within the socket in contrast to the action of CHX rinse which involves the socket and the whole oral cavity that reducing dental plaque and oral bacteria.

Haraji *et al*. (25)discussed the potential therapeutic effects of CHX on postoperative pain. They found that an intra-alveolar dose of CHX gel could significantly lower the pain for about 10% of potentially tolerable maximum pain (one rank) as compared with placebo gel. This result was in patients with and without Alveolar Osteitis (*P* ≤ .001).

In this study, we compared the percentage of pain grades assessed by (VAS) in both groups of patients who presented with AO. The results showed that grade 10 was the highest percentage for both of them and the level of pain was not affected by the form of CHX used.

The relation between technique of extraction and incidence of AO was estimated in this study. The percentage of AO following simple extraction was 12.3% which is within the average in the literature (1 - 30 %) (7). On the other hand, AO showed higher incidence with teeth extracted by root separation (29.2%). The difference in AO incidence between the percentage of AO after simple extraction and extraction by root separation was statistically significant (*p*=0.001). This result could be related to the greater trauma of alveolar bone in extraction by root separation.

The present study is one of very few studies which compared the efficiency of CHX rinse and gel in the prevention of AO post operatively. Also, the regimen of intra-alveolar CHX gel application was new, as it was applied directly after extraction and in day 3 rather than what was done by Hita-Iglesias *et al*. who applied CHX gel two times daily for a week. Additionally, this study is one of few studies performed to evaluate AO following simple extraction of molar teeth.

At the beginning of this study, we used to prescribe 0.12% CHX mouthwash for patients included. Unfortunately, we found some of them neglected to buy the mouthwash and were excluded from the study. Accordingly, we decided to give every patient a bottle of 0.12% CHX mouthwash to be sure that it will be used. Therefore, the application of 2.0% bioadhesive CHX gel post-operatively may produce an advantage over mouthwash, which is the avoidance of patient’s negligence regarding buying and using CHX mouthwash twice daily for a week. Further, intra-socket application of CHX gel by the dentist is helpful for patients who have difficulty to use CHX mouth wash like those with special needs and medically /mentally disabled patients.

In conclusion, direct intra-alveolar application of 0.2% CHX gel after extraction and in the third day post operatively insignificantly decreased the incidence of AO. Therefore, 0.2% CHX gel may be preferably used over 0.12% CHX mouthwash following molars extraction.
